# Maintenance of Health Behaviors: Exploring the Construct and Its Implications on Research, Education, and Practice

**DOI:** 10.1093/geroni/igaa057.3019

**Published:** 2020-12-16

**Authors:** Jaime Hughes

## Abstract

Modifying health behaviors, including diet, physical activity, sleep, and/or medication adherence, can have a range of positive effects on older adults’ overall health, function, and well-being. Although many evidence-based programs exist to support the initiation of health behavior changes, few address longterm maintenance. Emerging research suggests initiation and maintenance are distinct constructs, each requiring unique skills. Furthermore, maintaining health behaviors depends upon health promotion programs that are sustained, or continually delivered with high fidelity, at community and population levels. The objective of this symposium is to present findings from a series of research projects designed to investigate the concept of behavior change maintenance. Activities were supported by NIA Research Centers Collaborative Network (RCCN) funding and included community listening sessions plus an interdisciplinary think tank of national thought leaders. This symposium will begin with an overview of health behavior change, including the rationale for studying maintenance as a critical yet overlooked phase of successful behavior change (J. Hughes). A proposed conceptual model of maintenance will then be discussed, including constructs distinguishing maintenance from initiation (Raj). These introductory presentations will be followed by a discussion of multi-level barriers and facilitators related to maintenance on individual, community, and population levels (S. Hughes). The session will close with implications for research, education, and practice (Bettger).

